# Scraps

**Published:** 1898-09

**Authors:** 


### SCRAPS

PICKED UP BY THE ASSISTANT-EDITOR.

**Clinical Records** (24).—Sir Walter Scott was posting homewards through Yorkshire, and had occasion to stop at a smithy, one of his horses having cast a shoe. The smith and farrier, who proved to be a brother Scot, admitted that occasionally he practised on the human subject as well, but said that he worked with two simples only—"calomy and lodomy." When it was pointed out that these were keen weapons for unskilled hands to use, he did not seek to conceal the fact that occasional casualties happened among his Yorkshire patients; but added, turning to Sir Walter, as if on sure ground, "It will be long ere we make up for Flodden."

**Medical "Bits of Old Bristol"** (8).—Among the earlier water supplies of Bristol was that derived from a spring, which, in the 13th century, was in the orchard of the Priory of St. James. This orchard was situated on the north side of the present Maudlin Street, opposite where the Moravian Chapel now stands.

It is stated that towards the close of the century a supply from the spring was granted to the parish of All Saints, and that the pipe passed to Lewin's Mead, then over St. John's Bridge, and, going through Christmas Street, was carried along Broad Street to All Saints' Lane, at the corner of which the fountain stood. (Barrett's *History of Bristol*, pp. 458–9; Pryce's *History of Bristol*, p. 68; *The Pipes, Pumps, and Conduits of Bristol*, p. 21; *Bristol Past and Present*, vol. i., p. 139.) But there is grave reason to doubt the accuracy of some of these statements, and I hope that Mr. Latimer will enable me in a future number to give exact information concerning this portion of the water-supply.

In 1601 the conduit was taken to the south-western corner of the church, at which spot it was erected in a style more ornamental than when Leland saw it about the middle of the sixteenth century. The picture of All Saints' Church which Barrett gives and which is reproduced in *Bristol Past and Present* (ii. 72) shows the fountain as it appeared in its renovated condition. On the building next the church can be seen even now indications of the position of the 1601 structure. The water can still be obtained; but it now comes through a modern tap, which is placed nearer the church door.

Some of the above-named authorities say that from the All Saints' pipe a supply was taken to the parish of St. Nicholas. At the north-west corner of old Bristol Bridge stood a fountain. This was taken down when the 1762 bridge was built and re-erected, Mr. Latimer says, "on the Back." (*Annals of Bristol in the Eighteenth Century*, p. 353.) I am not able to indicate the situation it there occupied, and I do not know if any remains of it exist. Mr. Latimer (*op. cit.*, p. 141) describes the position of its predecessor also as "on the Back."

**Medical Philology** (XXVII.).—The early therapeutists, in paying special attention to the dietetic treatment of phthisis, recommended that which is described in the *Catholicon* as "a Culice, *morticium*," and in Pynson's 1499 edition of the *Promptorium* as "Colysshe, disshe mete."

Mr. Way in his note says that "in the collection of Recipes, dated 1381, printed with the Forme of Cury, will be found one 'for to make a Colys,' which was a sort of invigorating chicken broth." Mr. Herrtage's note states that "Andrew Boorde, in his Dyetary (E. E. Text Soc., ed. Furnivall), p. 264, speaks of 'Caudeles made with hempe sede, and *collesses* made of shrympes,' which, he says, 'doth comforte blode and nature.' . . . Directions for 'a *coleise* of a cocke for a weake body that is in a consumption,' are given by Cogan, Haven of Health, 1612, p. 131. 'Broth or collyse, *pulmentarium*.' Huloet." John Russell, in his *Boke of Nurture*, written about 1460, in a list of "Sewes on fishe dayes," mentions "colice of pike, shrympus or p*er*che." Dr. F. J. Furnivall has edited Russell's work for the Early English Text Society. On p. 172 he gives the following note:

"Cullis (in Cookery) a strained Liquor made of any sort of dress'd Meat, or other things pounded in a Mortar, and pass'd thro' a Hair-sieve: These Cullises are usually pour'd upon

Messes, and into hot Pies, a little before they are serv'd up to Table. Phillips . . . Shrimps are of two sorts, the one crookbacked, the other straitbacked: the first sort is called of Frenchmen *Caramots de la santé*, healthful shrimps; because they recover sick and consumed persons; of all other they are most nimble, witty, and skipping, and of best juice. *Muffett*, p. 167. In cooking them, he directs them to be 'unscaled, to vent the windiness which is in them, being sodden with their scales; whereof lust and disposition to venery might arise,' p. 168."

Nares, in his *Glossary*, defines cullis as "a very fine and strong broth, strained and made clear for patients in a state of great weakness." He gives the receipt from the *Haven of Health*, saying that it "in many respects is so curious, that I am tempted to insert the whole of it":

"If you list to still [distil] a cocke for a weak body, that is in a consumption through long sicknesse or other causes, you may doe it well in this manner. Take a red cocke, that is not old, dresse him and cut him in quarters, and bruse all the bones, then take the rootes of fennell, parcely, and succory, violet leaves, and borage, put the cocke into an earthen pot which is good to stew meates in, and between every quarter lay of the rootes and herbes, corans, whole mace, anise seeds, liquorice being scraped and slyced, and so fill up your pot. Then put in halfe a pint of rose water, a quart of white wine or more, two or three dates made cleane and cut in peices, a few prunes and raysons of the sunne, *and if you put in certain peeces of gold, it will be the better, and they never the worse*, and so cover it close, and stop it with dough, and set the pot in seething water, and let it seeth gently for the space of twelve houres, with a good fire kept still under the brasse pot that it standeth in, and the pot kept with liquor so long. When it hath stilled so many houres, then take out the earthen pot, open it, streine out the broth into some cleane vessel, and give thereof unto the weake person morning and evening, warmed and spiced, as pleaseth the patient. In like manner you may make a *coleyse* of a capon, which some men like better."

Nares adds that "Brown, in his Pastorals, tells us of a *cullis* mixed with still more costly ingredients:

"To please which Orke her husband's weakned peece
Must have his *cullis* mixt with *ambergreece*,
Phesant and partridge into jelly turn'd,
Grated with *gold* sev'n times refin'd and burn'd,
With *dust of Orient pearle*, richer the east
Yet ne're beheld: (O Epicurian feast!)
This is his breakfast."—*Brit. Past.*, B. ii., S. 3.

Although the preparation is not mentioned by Shakspere, it is referred to by many writers of his period. Nares quotes some of them. Its use in phthisis is mentioned by Lyly in *Euphues*, and in much the same terms in one of his plays:

"He that melteth in a consumption is to be recured by colices, not conceits."
*Campaspe* (ed. Fairholt), III. v.

Middleton has several references to it, two of which confirm the costliness of the prescription given in *Britannia's Pastorals*:

"Let gold, amber, and dissolved pearl, be common ingrediencies, and that you cannot compose a cullice without 'em."—*A Mad World, my Masters* (ed. Bullen), II. vi. 49—51.

"*Antonio.* Give order that two cocks be boil'd to jelly.
*Gasparo.* How? two cocks boil'd to jelly?
*Antonio.* Fetch half an ounce of pearl.
*Gasparo.* This is a cullis
For a consumption."
*The Witch* (ed. Bullen), II. i. 11—14.

Beaumont and Fletcher (*Captain*, I. iii.) also mention gold as one of the ingredients of a cullis. On the question of potable gold, I made a few comments when referring, in the *Journal* of June, 1894, to Chaucer's Doctor of Physic.

That the cullis was not used as a restorative for the phthisical only, may be gathered from the comedy of *Lingua*, a play first printed in 1607, but probably written in Elizabeth's reign. Appetitus, the "parasite," remarks: "I lie with the ladies all night, and that's the reason they call for cullies and gruellies so early before their prayers."—Dodsley's *Old English Plays*, ed. Hazlitt, vol. ix., p. 366.

The word is to be met with in some modern cookery books. Many ways in which it was spelled other than those I have given can be seen in the *New English Dictionary*. Remembering the Latin *colare*, to strain, and that we still have "*Colander*, a strainer," we see that the etymology is clear. Cotgrave gives, "Coulis: m. *A cullis, or broth of boyled meat strained; fit for a sicke, or weake bodie.*"

Middleton (*Family of Love*, III. ii.) and Fletcher (*Nice Valour*, III. i.) use the word metaphorically in reference to its restorative power and to the necessity of pounding or pressing the meat used.

